# Hematological and Histopathological Effects of Subacute Aconitine Poisoning in Mouse

**DOI:** 10.3389/fvets.2022.874660

**Published:** 2022-04-05

**Authors:** Hao Lu, Li Mei, Ziyu Guo, Kexin Wu, Yunhao Zhang, Shiyu Tang, Yiru Zhu, Baoyu Zhao

**Affiliations:** ^1^College of Veterinary Medicine, Northwest A&F University, Xianyang, China; ^2^College of Landscape and Architecture and Art, Northwest A&F University, Xianyang, China

**Keywords:** aconitine, subacute poisoning, hematological indices, histological changes, mice

## Abstract

Aconitine is the principal toxic ingredient of *Aconitum*, which can cause systemic poisoning involving multiple organs and systems after animal ingestion. The purpose of this study was to investigate the effects of aconitine on hematological indices and histological changes in mice. One hundred twenty mice were divided into a control group (normal saline), low-dose group (0.14 μmol/L), middle-dose group (0.28 μmol/L) and high-dose group (0.56 μmol/L), which were continuously lavaged for 30 days. The blood of 10 mice were collected randomly and analyzed by group at the 10th, 20th, and 30th days, and some tissues were collected and stained with hematoxylin-eosin to observe histological changes at the 30th day. Compared with the control group, the organ coefficient (%) of liver, spleen, lungs, and brain of the high-dose group were significantly increased (*p* < 0.05 or *p* < 0.01). WBC and Gran initially decreased and then increased in each poisoning group, with significant differences in the high-dose group (*p* < 0.05 or *p* < 0.01). RBC, HGB, HCT, and PLT decreased continuously in all groups except the low-dose group at the 20th and 30th days (*p* < 0.05 or *p* < 0.01). Moreover, BUN, ALT and AST increased in each poisoning group, in comparison with the control group, with significant differences except for the low-dose group (*p* < 0.05 or *p* < 0.01). CRE initially increased and then decreased, the TP and ALB decreased, with significant differences observed in the high-dose and middle-dose groups (*p* < 0.05). All the mice in the poison-treated groups showed varying degrees of histopathological changes such as degeneration and necrosis of tissues, especially heart and cerebellum. Our data suggest that different doses of aconitine have remarkable effects on hematological and histopathological changes in mice, in a significant time and dose-effect relationship.

## Introduction

Aconitum L. is a family of 1-year-to-perennial herbaceous plants within the *Ranunculaceae* that is both medicinal and poisonous. The plants are widely distributed in temperate zones of Northern Hemisphere, mainly in Asia, Europe, and North America. China is one of the countries with richest species resources of aconitum in the world, mainly distributed in Sichuan, Yunnan, Tibet and other provinces in Southwest China (1). There are more than 200 species of Aconitum in China, including *Aconitum leucostomum, Aconitum kusnezoffii, Aconitum carmichaelii, Aconitum coreanum*, and *Aconitum pendulum* (2). As a result of climate change and overgrazing, natural grasslands have degenerated, leading to a rapid increase in poisonous plants including aconitum, which has caused huge economic losses in animal husbandry in the grasslands. Animals can be poisoned after ingesting Aconitum, with clinical symptoms of salivation, vomiting, diarrhea, dyspnea, sensory paralysis, and eventually death from cardiopulmonary failure (3).

**Figure 1:**
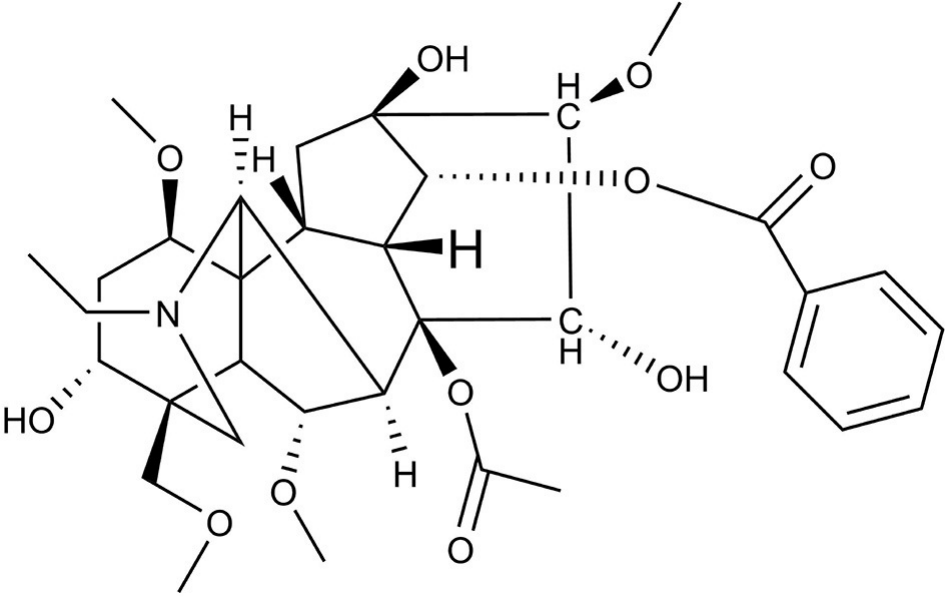
The chemical structure of aconitine.

Studies have indicated that aconitum has pharmacological activities including analgesic (4), cardiac (5), anti-aging (6), anti-inflammatory (7), anti-tumor (8) and anti-viral activity (9). Aconitum contains more than 90 kinds of alkaloids, including aconitine, hypaconitine, and mesaconitine (10, 11). Aconitine, a diterpene alkaloid, is the principal toxic ingredient of Aconitum (Figure 1) (11). The reported LD_50_ values of Aconitine in mice were 1.8 mg/kg orally and 0.308 mg/kg intraperitoneally (12). Zhou et al. reported Aconitum was highly toxic including cardio-toxicity and neurotoxicity, embryo toxicity and renal toxicity. Its toxicological mechanism has been shown in its effect on voltage-dependent sodium channels, release of neurotransmitters and changes in receptors, promotion of lipid peroxidation and cell apoptosis in heart, liver and other tissues (13). The toxicological research on aconitine has mainly focused on acute cardiotoxicity (14–16) and neurotoxicity (17–19). Peng et al. investigated the neurotoxic effects and underlying mechanisms of aconitine on cerebral cortex neuron cells prepared from neonatal SD (Sprague-Dawley) rats. They found aconitine may damage neuron cells through its inhibition of Na^+^-K^+^-ATP activity and neurotransmitters in the cells, which resulted in injuries to cell morphology and function (20). Subsequently, Gao et al. investigated the apoptotic effects of aconitine in H9c2 cardiac cells and concluded that aconitine might induce apoptosis through mitochondria-mediated signaling pathways (21).

However, there have been no systematic studies reported on the hematological and histopathological influence of the subacute administration of aconitine. Thus, our study was conducted to investigate the hematological indices and histopathological changes due to aconitine treatments in experimental animal models, to provide an important theoretical basis for the research on the toxic mechanism of aconitine. These results may contribute valuable information to toxicology.

## Materials and Methods

### Reagents

Aconitine (purity 98.86%) was purchased from Chengdu Mansite Bio-Technology Co.,Ltd. (MUST-17022206, China). One percent dilute hydrochloric acid, 1 M HCL, 1 M NaOH and normal saline were from Shaanxi Biostar Pharmaceutical Co., Ltd.

### Animals

Four-week-old Kunming mice weighing approximately 20 g were used in all the experiments. The mice were purchased from Chengdu Dashuo Experimental Animal Co., Ltd. The experimental procedures were in accordance with the Ethical Principles [Animal (Scientific Procedures) Act 2012] in Animal Research adopted by the China College of Animal Experimentation and were approved by the College of Veterinary Medicine-Northwest A&F University. Since the mice were obtained from a commercial source, a consent to participate statement is not required.

### Study Design

#### Establishment of a Subacute Poisoning Model of Aconitine in Mice

Animals were randomly assigned into four experimental groups, consisting of 30 (15 male and 15 female) animals per group. One hundred twenty mice were assigned to either a control group (normal saline) or low dose group (0.14 μmol/L), middle dose group (0.28 μmol/L) and high dose group (0.56 μmol/L), After a week-long adaptation period in a room with controlled temperature (21 ± 1°C) and lighting (12 h light/12 h dark), relative humidity maintained between 40 and 70%, adequate commercial rat chow and tap water are given during feeding. Mice were continuously lavaged for 30 days. Aconitine was dissolved in 1% dilute hydrochloric acid then add normal saline, adjust PH to 7 before administered.

#### Organ Coefficient Assessment

All mice were euthanized via cervical dislocation by well-trained operators. The cerebrum, cerebellum, heart, liver, spleen, lungs, and kidneys of 10 mice were collected randomly in each group at the 10th, 20th, and 30th days. The attached fat and fascia were carefully removed and weighed and the organ coefficient (%) was calculated according to the following formula: organ coefficient (%) = (organ weight/body weight) ×100%.

#### Hematological Assessment

Blood was collected when mice were sacrificed for each group at 10th, 20th, and 30th days evenly. Collected 1–2 drop of blood in EDTA-2K anticoagulation tube to determine white blood cells (WBC), red blood cells (RBCs), hemoglobin (HGB), hematocrit (HCT), blood platelets (PLTs) and granulocyte (Gran) using an automatic Blood Cell Analyzer, BC-2800vet (Mindray, China). The blood samples were centrifuged 15 min for collect the serum and measure the activity of alanine aminotransferase (ALT) and aspartate aminotransferase (AST) and the content of blood urea nitrogen (BUN), total protein (TP), albumin (ALB) and creatinine (CRE) using the Hitachi-7180 automatic biochemical analyzer (Hitachi, Japan).

#### Histopathological Preparation

All tissues were removed and fixed in 10% formaldehyde at room temperature. The tissue samples were then dehydrated and embedded in paraffin according to standard histological procedures. Serial cross-sections of 3–5 μm were prepared from each organ. The sections were mounted and stained with hematoxylin-eosin. The histopathological changes in the major organs were observed with a fluorescent vertical microscope (10 ×40 times).

#### Statistical Analysis

Results are expressed as mean ± standard deviation. All data were analyzed in SPASS 20.0 using one-way analysis of variance (ANOVA) followed by Duncan's test for multiple comparisons. *p* < 0.05 was considered significant, and *p* < 0.01 was considered extremely significant.

## Results

### Effect of Aconitine on the Body Weight in Mice

**Figure 2:**
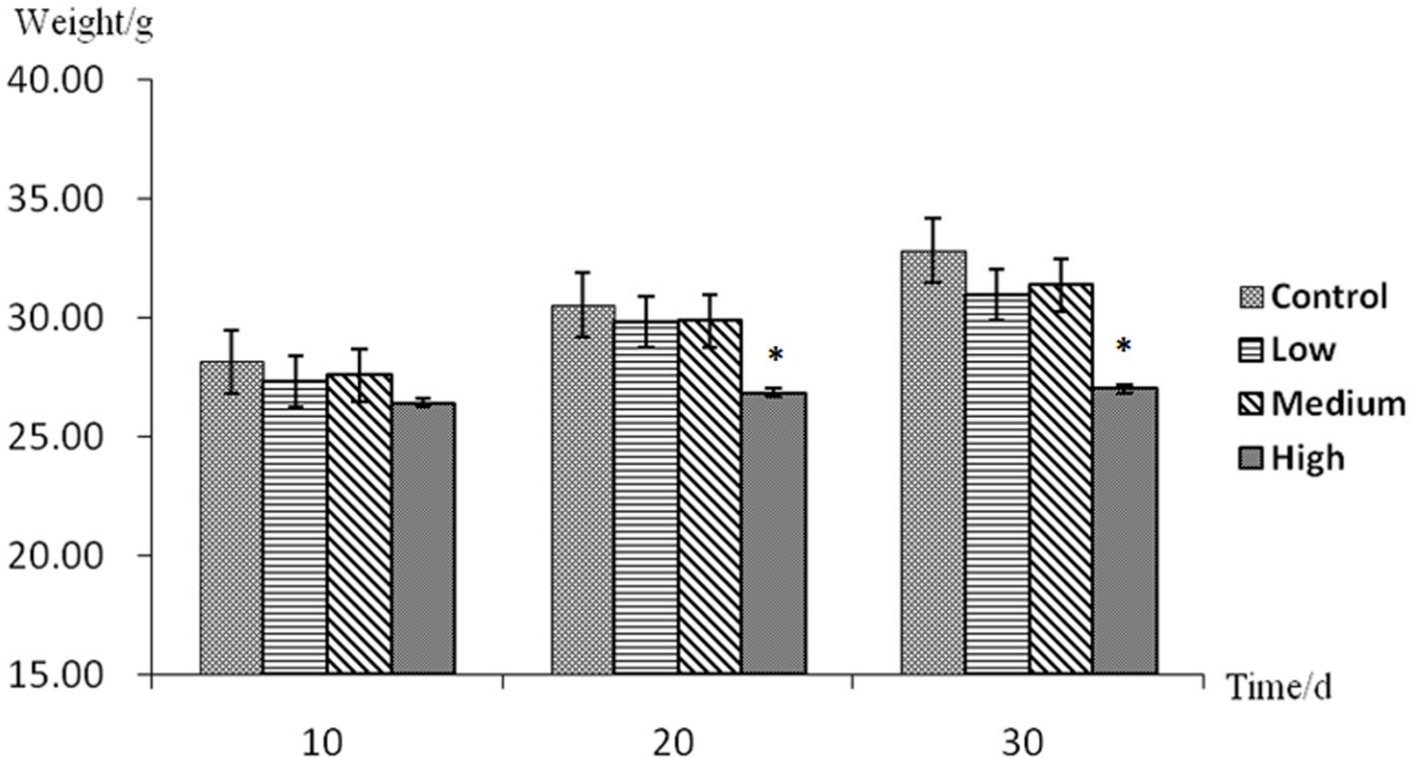
Body weight assessment with no mark in the same row differ insignificantly (*p* > 0.05); while with “*” differ significantly (*p* < 0.05).

The average weight growth trends in all experimental groups were lower than the control group (Figure 2). At the 10th day, there was no significant difference between the experimental groups and the control group (*p* > 0.05). At the 20th and 30th days, the low-dose group and the middle-dose group mice were not statistically significantly different from the control (*p* > 0.05). However, there was a significant difference between the high-dose group and the control group (*p* < 0.05).

### Effect of Aconitine on the Organ Coefficient (%) in Mice

Compared with the control group, the weight of heart and kidneys increased in each poisoning group while there was no significant change in organ coefficient (%) during administration. The weight of the liver in each poisoning group increased initially and then decreased, with the highest change in the high dose group. At the 20th day after treatment, the liver organ coefficient (%) in the high-dose group was significantly reduced compared with the control group (*p* < 0.01). At the 30th day after treatment, the organ coefficient (%) of liver in the high-dose group decreased, which was significantly different from that of the control group (*p* < 0.05). The organ coefficient (%) of spleen was significantly reduced. At the 30th day after treatment, the difference between the high-dose group and the control group was highly significant (*p* < 0.01). The organ coefficient (%) of lungs increased with increasing dose and length of treatment, and the organ coefficient (%) of lungs in the high dose group was significantly different from that in the control group at the 30th day (*p* < 0.05). The organ coefficient (%) of the brain was significantly lower than that of the control group. At the 30th day after treatment, there was a significant difference between the control and the high dose group (*p* < 0.05). Cerebellar organ coefficient (%) showed no significant differences. The result of tissue coefficient (%) can be shown in Table 1.

**Table 1:** Changes of organ coefficient in mouse with subacute aconitine poisoning.

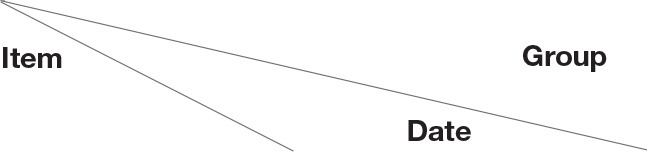	**Control**	**Low-dose**	**Middle-dose**	**High-dose**
Heart	10th day	0.62 ± 0.09	0.61 ± 0.09	0.60 ± 0.11	0.59 ± 0.07
	20th day	0.62 ± 0.09	0.63 ± 0.05	0.60 ± 0.04	0.62 ± 0.06
	30th day	0.62 ± 0.08	0.62 ± 0.11	0.63 ± 0.11	0.68 ± 0.16
Liver	10th day	5.53 ± 0.33	5.26 ± 0.49	5.12 ± 0.45	5.44 ± 0.65
	20th day	5.80 ± 0.30	5.49 ± 0.37	5.47 ± 0.13	5.09 ± 0.20**
	30th day	5.62 ± 0.26	5.39 ± 0.40	5.09 ± 0.44	4.98 ± 0.85*
Spleen	10th day	0.53 ± 0.32	0.41 ± 0.07	0.44 ± 0.10	0.33 ± 0.07
	20th day	0.38 ± 0.03	0.37 ± 0.07	0.36 ± 0.07	0.29 ± 0.10
	30th day	0.39 ± 0.07	0.39 ± 0.04	0.36 ± 0.05	0.28 ± 0.05**
Lung	10th day	0.91 ± 0.38	0.79 ± 0.15	0.78 ± 0.23	0.77 ± 0.09
	20th day	0.77 ± 0.10	0.71 ± 0.09	0.78 ± 0.11	0.79 ± 0.10
	30th day	0.74 ± 0.09	0.76 ± 0.10	0.77 ± 0.20	0.92 ± 0.10*
Kidney	10th day	1.51 ± 0.28	1.39 ± 0.21	1.47 ± 0.20	1.67 ± 0.42
	20th day	1.48 ± 0.24	1.57 ± 0.29	1.57 ± 0.23	1.58 ± 0.22
	30th day	1.66 ± 0.20	1.65 ± 0.28	1.67 ± 0.20	1.71 ± 0.32
Cerebrum	10th day	1.20 ± 0.24	1.19 ± 0.11	1.27 ± 0.14	1.32 ± 0.17
	20th day	1.09 ± 0.18	1.12 ± 0.16	1.10 ± 0.20	1.12 ± 0.12
	30th day	1.10 ± 0.26	1.11 ± 0.14	1.19 ± 0.18	1.33 ± 0.07*
Cerebellum	10th day	0.50 ± 0.07	0.55 ± 0.07	0.51 ± 0.12	0.54 ± 0.05
	20th day	0.44 ± 0.09	0.45 ± 0.12	0.46 ± 0.10	0.47 ± 0.12
	30th day	0.45 ± 0.10	0.45 ± 0.08	0.45 ± 0.10	0.53 ± 0.06

n = 10, the data in table are M ± SD, means with no mark in the same row differ insignificantly (p > 0.05); while with “*” differ significantly (p < 0.05) with “**” differ extremely significant.

### Effect of Aconitine on the Hematological Indexes in Mice

As the dose and time of administration increased, WBC and Gran decreased initially and then increased in each poisoning group. RBC, HGB, HCT and PLT decreased continuously in all groups except for the low-dose group at the 20th and 30th days. At the 10th day after treatment, RBC, HGB, WBC and PLT revealed no significant difference between the experimental groups (*p* > 0.05), while the content of HCT decreased and Gran increased significantly in the high-dose group (*p* < 0.05). After the 20th day of lavage, RBC, HGB, HCT levels in the middle-dose and high-dose group were significantly decreased compared with the controls (*p* < 0.05). Gran levels in the middle-dose group were significantly increased compared with the control group (*p* < 0.05). PLT levels in the high-dose group were highly significantly decreased (*p* < 0.01). WBC in treatment groups was not significantly different. At the 30th day, the level of RBC, HGB, HCT, PLT decreased significantly except for the content of HGB and PLT in the low-dose group. WBC levels in the low-dose and high-dose groups were significantly decreased (*p* < 0.05). The content of Gran in the high-dose group increased significantly (*p* < 0.05) (Table 2).

**Table 2:** Characterization of physiological indexes in blood with subacute acontine poisoning.

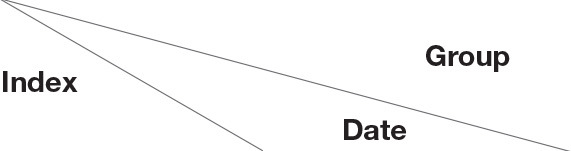	**Control**	**Low-dose**	**Middle-dose**	**High-dose**
RBC (10^12^/L)	10th day	8.972 ± 0.643	8.825 ± 0.394	8.780 ± 0.760	8.533 ± 0.466
	20th day	8.913 ± 0.428	8.640 ± 0.370	8.253 ± 0.632*	7.852 ± 0.615**
	30th day	8.901 ± 0.364	8.044 ± 0.418*	7.623 ± 0.955**	6.896 ± 0.738**
HGB (g/L)	10th day	142.167 ± 4.956	141.167 ± 9.368	139.000 ± 9.859	135.167 ± 5.601
	20th day	142.500 ± 3.017	139.500 ± 1.871	136.500 ± 3.619**	132.333 ± 2.251**
	30th day	142.429 ± 8.979	137.429 ± 3.952	133.286 ± 3.498**	123.143 ± 4.140**
HCT (%)	10th day	45.750 ± 3.112	44.283 ± 2.424	43.800 ± 2.184	41.700 ± 1.364**
	20th day	45.383 ± 1.446	42.950 ± 2.455	41.417 ± 1.646*	38.067 ± 4.004**
	30th day	45.314 ± 2.968	40.086 ± 4.983**	38.086 ± 2.180**	33.886 ± 3.292**
WBC (10^9^/L)	10th day	4.033 ± 0.561	3.733 ± 0.967	4.383 ± 1.225	4.883 ± 0.527
	20th day	4.083 ± 0.799	4.417 ± 1.372	4.850 ± 0.689	3.983 ± 1.030
	30th day	4.071 ± 0.386	4.929 ± 0.150*	3.700 ± 0.823	2.614 ± 1.221**
Gran# (10^9^/L)	10th day	2.017 ± 0.232	2.167 ± 0.216	2.383 ± 0.366	2.583 ± 0.462**
	20th day	1.983 ± 0.264	2.283 ± 0.331	2.467 ± 0.505*	2.217 ± 0.232
	30th day	2.043 ± 0.369	2.457 ± 0.479	2.314 ± 0.677	1.486 ± 0.406*
PLT (10^9^/L)	10th day	797.167 ± 95.613	784.500 ± 64.302	763.333 ± 39.343	746.500 ± 19.024
	20th day	793.000 ± 66.903	769.333 ± 34.168	738.667 ± 55.558	683.000 ± 69.745**
	30th day	795.000 ± 104.916	747.000 ± 52.612	696.571 ± 50.964*	619.286 ± 52.832**

n = 10, the data in table are M ± SD, means with no mark in the same row differ insignificantly (p > 0.05); while with “*” differ significantly (p <0.05) with “**” differ extremely significant.

Of the biochemical indices, ALT, AST, and BUN increased, showing a clear dose-effect relationship. The content of CRE in the low-dose group first decreased and then increased, however, it showed a rising trend in the middle-dose group and high-dose group, while TP, ALB content showed a decreasing trend. After 10 days of dosage, the activity of ALT and AST in treatment groups increased but the level of ALB decreased. Except for the low dose group, there was a significant increased compared with the controls (*p* < 0.05). The content of BUN increased while TP decreased, and it significantly differed in high-dose group (*p* < 0.01), however the content of CRE was not significantly different (*p* > 0.05). At the 20th day, the activity of AST significantly increased in treatment groups (*p* < 0.01). The level of ALT, BUN, and CRE in the high-dose and middle-dose groups increased while the content of TP and ALB decreased significantly (*p* < 0.05). At the 30th day, all indices in aconitine-treated mice were statistically different from with the controls (Table 3).

**Table 3:** Biochemical marker characterization of aconitine-treated mouse.

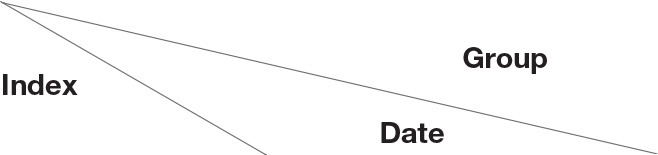	**Control**	**Low-dose**	**Middle-dose**	**High-dose**
ALT(U/L)	10th day	30.20 ± 0.77	32.83 ± 1.08	35.42 ± 1.84**	37.78 ± 0.68**
	20th day	31.05 ± 0.49	35.58 ± 1.67	39.67 ± 2.34**	44.43 ± 2.65**
	30th day	30.85 ± 0.94	37.18 ± 2.65*	42.97 ± 1.30**	49.95 ± 1.41**
AST(U/L)	10th day	96.50 ± 1.15	99.72 ± 0.81	111.47 ± 2.84**	123.08 ± 3.86**
	20th day	94.55 ± 0.56	109.00 ± 2.63**	116.20 ± 5.12**	137.32 ± 3.74**
	30th day	96.22 ± 1.65	116.60 ± 1.72**	130.12 ± 3.37**	152.52 ± 3.55**
BUN(mmol/L)	10th day	5.39 ± 0.17	5.74 ± 0.16	6.00 ± 0.17	6.40 ± 0.23**
	20th day	5.34 ± 0.11	5.86 ± 0.40	6.27 ± 0.27*	7.19 ± 0.19**
	30th day	5.51 ± 0.23	6.22 ± 0.24*	6.63 ± 0.24**	8.17 ± 0.22**
CRE(mmol/L)	10th day	6.55 ± 0.36	6.30 ± 0.40	6.83 ± 0.32	7.33 ± 0.47
	20th day	7.02 ± 0.24	7.30 ± 0.21	8.83 ± 0.34**	9.70 ± 0.34**
	30th day	6.62 ± 0.35	8.10 ± 0.23**	9.43 ± 0.20**	10.85 ± 0.52**
TP(g/L)	10th day	57.68 ± 1.48	57.08 ± 1.37	55.25 ± 1.86	50.47 ± 1.63**
	20th day	58.60 ± 1.40	55.40 ± 1.45	52.22 ± 1.03**	45.17 ± 2.01**
	30th day	59.28 ± 1.60	52.68 ± 1.15**	47.88 ± 1.78**	38.83 ± 1.98**
ALB(g/L)	10th day	21.87 ± 0.81	21.25 ± 0.51	19.03 ± 0.82*	17.57 ± 1.08**
	20th day	21.92 ± 0.67	20.88 ± 0.45	17.90 ± 0.86**	15.55 ± 0.42**
	30th day	22.37 ± 0.70	19.63 ± 0.60**	16.08 ± 0.29**	12.85 ± 0.76**

n = 10, the data in table are M ± SD, means with no mark in the same row differ insignificantly (p > 0.05); while with “*” differ significantly (p <0.05) with “**” differ extremely significant.

### Effect of Aconitine on the Histopathology in Mice

#### Cerebrum

**Figure 3:**
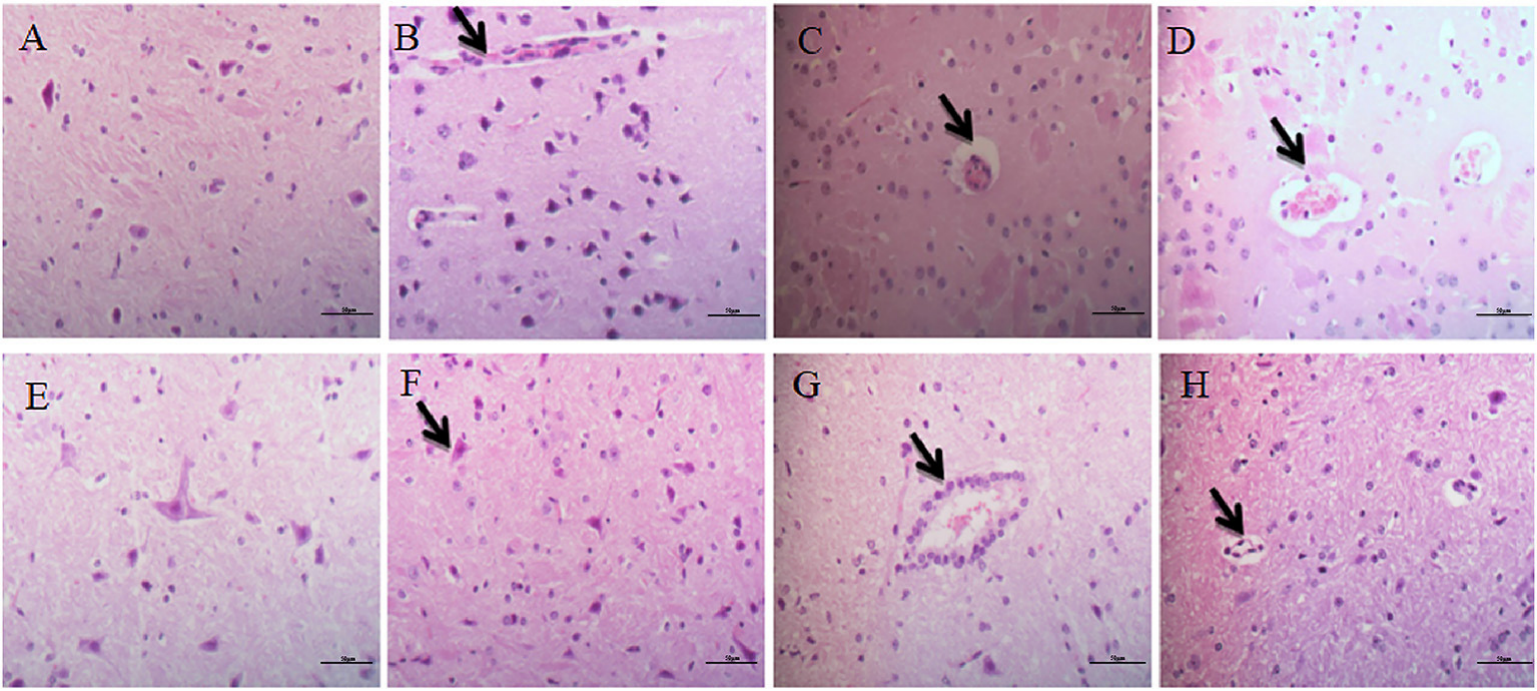
Histopathological alterations in the brain. **(A–D)** Represent changes in the cerebrum of control group, low-dose group, middle-dose group, high-dose group (×400); **(E–H)** represent changes in the cerebellum of control group, low-dose group, middle-dose group, high-dose group (×400). The black arrow masks edema and slight neuronophagia **(B–D)**, vacuolization of purkinje cells **(F–H)**.

The cerebrum structure of the control group was complete and showed no significant alterations (Figure 3A). There was no significant changes in the intercellular substance of the cerebral cortex in the low-dose group compared to the control (Figure 3B). The pathological changes in the middle-dose group displayed minimal edema and slight neuronophagia (Figure 3C). In the high-dose group, some cortical cells in the cerebrum were swollen, degenerated and necrotized. There was noticeable neuronophagia. The intercellular substance of cerebral cortex was loose. Aconitine caused blood vessels in the brain to dilate and fill with red blood cells (Figure 3D).

#### Cerebellum

Examination of the cerebellar cortex in the control group revealed no marked changes (Figure 3E). The low-dose and middle-dose groups showed slight vacuolization of Purkinje cells (Figures 3F,G). In the high-dose group, Purkinje cells were swollen, degenerated and necrotized. There was obvious cell vacuolation in the cytoplasm (Figure 3H).

#### Heart

**Figure 4:**
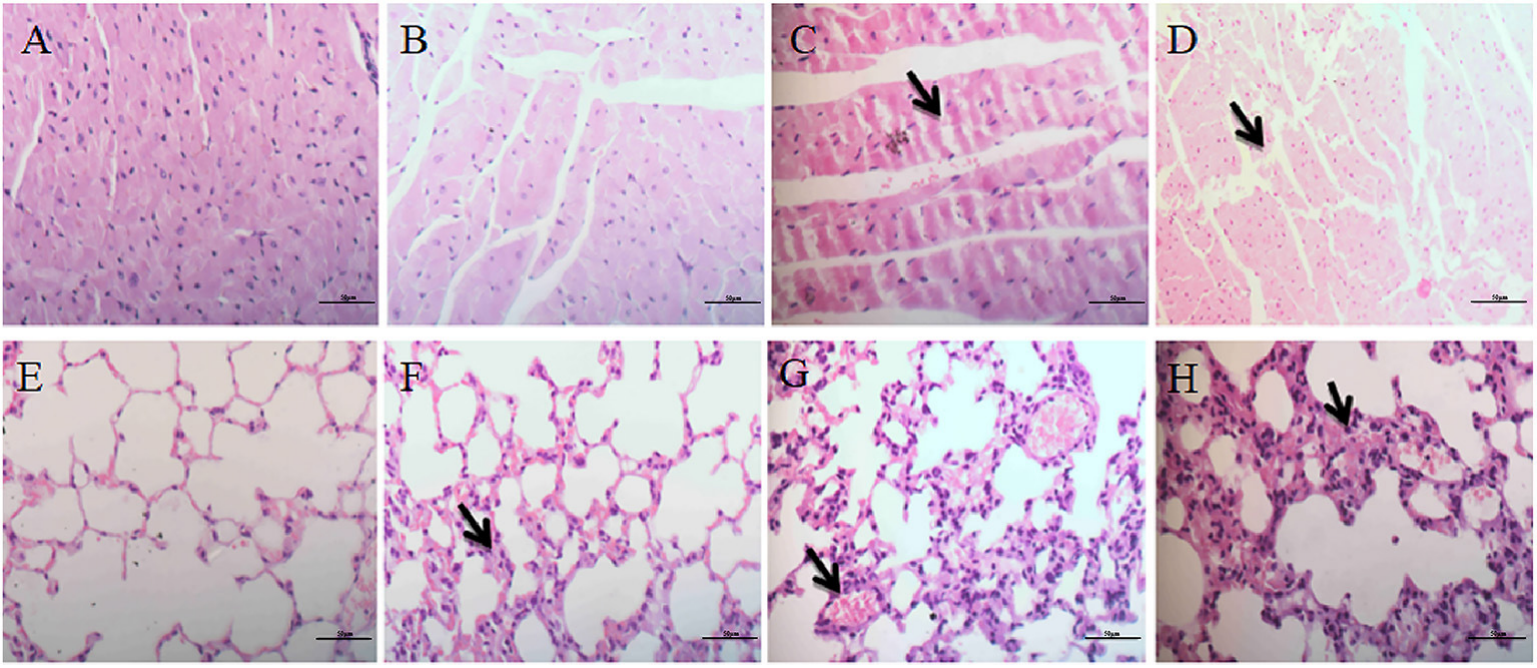
Histopathological effects on heart and lungs. **(A–D)** Represent changes in the heart of control group, low-dose group, middle-dose group, high-dose group (×400); **(E–H)** represent changes in the lungs of control group, low-dose group, middle-dose group, high-dose group (×400). The black arrow masks swelling and necrosis and fracture of the myocardial fibers **(C,D)**, congestion and hemorrhage and inflammatory cell infiltration of the alveolar spaces **(F–H)**.

In the control group, the myocardial fibers were arranged regularly and neatly with no obvious pathological changes in the interstitium (Figure 4A), while no obvious lesions were seen in the low-dose group (Figure 4B). In the medium and high dose groups, the myocardial fibers were swollen, necrotic and fractured. At the same time, interstitial connective tissues exhibited cell proliferation (Figures 4C,D).

#### Lungs

No significant pathological changes were observed in the lungs of the control group (Figure 4E). The mice in the low-dose group had mild congestion in the lungs (Figure 4F). In the middle-dose group, a small number of alveolar walls were thickened, and heavier hyperemia and hemorrhage were observed (Figure 4G). In the high-dose group, alveolar walls were thickened and merged with each other, and inflammatory cell infiltration was observed in the alveolar spaces. The interstitial capillaries were congested and the epithelial cells of bronchiole were abscised (Figure 4H).

#### Liver

**Figure 5:**
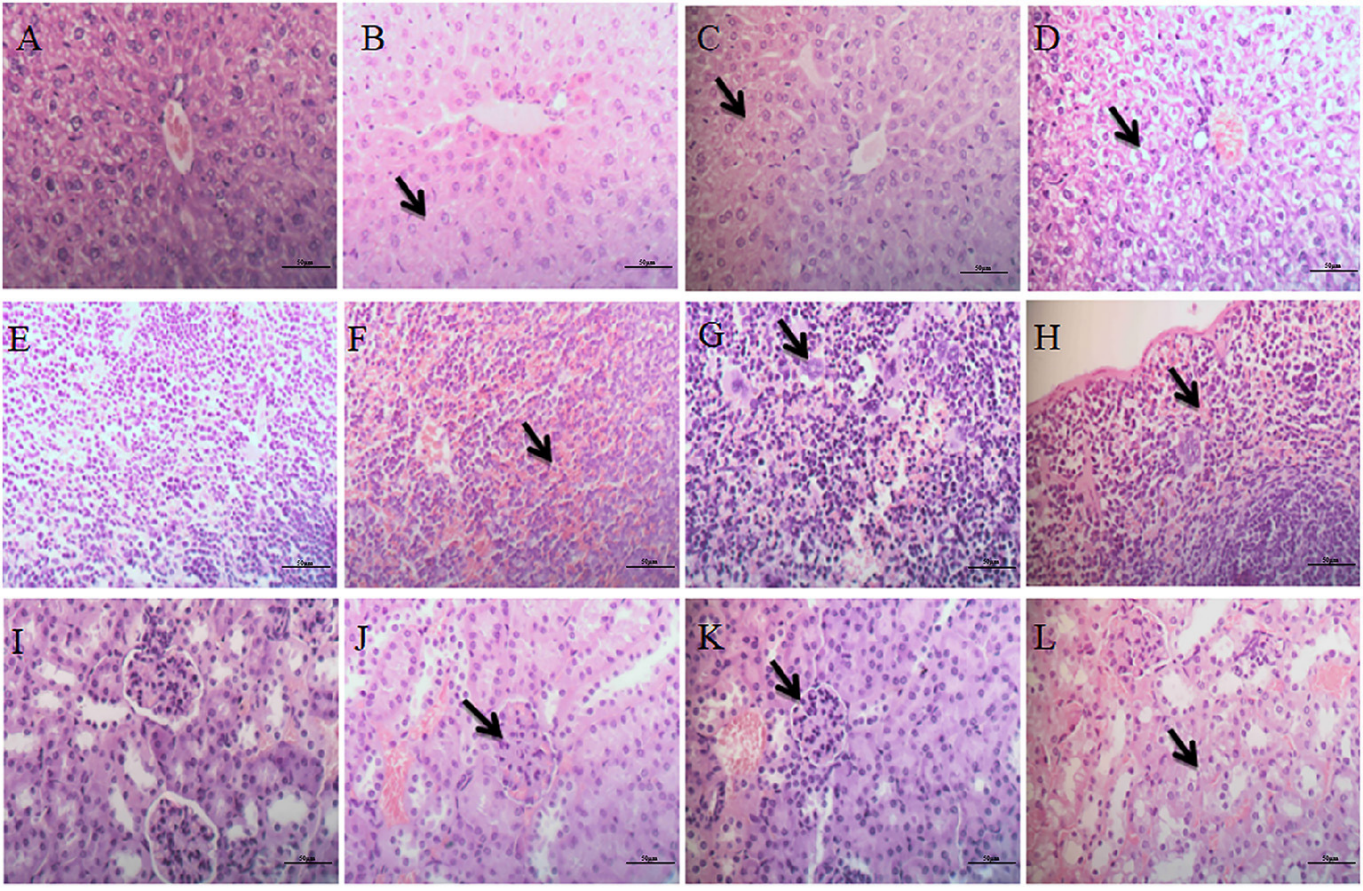
Histopathological examination of liver, spleen and kidneys. **(A–D)** Represent changes in the liver of control group, low-dose group, middle-dose group, high-dose group (×400); **(E–H)** represent changes in the spleen of control group, low-dose group, middle-dose group, high-dose group (×400). **(I–L)** Represent changes in the kidneys of control group, low-dose group, middle-dose group, high-dose group (×400). The black arrow masks the disorganized hepatic cord and cytoplasm vacuolization **(B–D)**, varying the number of megakaryocytes **(F–H)**, Interstitial hyperemia and hemorrhage, flocular degeneration of renal tubular epithelial cells **(J–L)**.

The hepatic cords of the control group were arranged neatly, and the structure of the portal area was normal (Figure 5A). In the low-dose group, the central vein was slightly congested, and the hepatocyte cytoplasm was slightly vacuolized (Figure 5B). In the middle-dose group and high-dose group, histopathological changes indicated the presence of hyperaemia in hepar, the hepatic cord in hepar was disorganized, and swelling, and granular degeneration was noted in the cytoplasm (Figures 5C,D). Furthermore, we observed hepatic sinusoid stenosis and cytoplasm vacuolization in the high-dose group (Figure 5D).

#### Spleen

The spleen in the control group showed no obvious pathological changes (Figure 5E). Mild lesions were seen in the low-dose group, and a few megakaryocytes were visible (Figure 5F). There were significantly more megakaryocytes in the middle-dose group than other groups (Figure 5G). In the high-dose group, splenic corpuscles were broken or missing, red pulp widened and white pulp atrophied, splenic sinusoid extended with hyperemia, and a large number of megakaryocytes were seen in the spleen (Figure 5H).

#### Kidneys

The glomeruli and renal tubules were histologically normal in the control group, and the interstitial tissue was dense (Figure 5I). In the low-dose group, there was no significant alteration in glomeruli or renal tubules, but interstitial congestion and hemorrhaging were obvious (Figure 5J). The glomeruli in the middle-dose group had no obvious lesions, while the cytoplasm of renal tubular epithelial cells showed flocular degeneration, and the nucleus was difficult to identify. The tubular epithelium of the distal tubule of the renal tubule was exfoliated with exudate, and the interstitial hyperemia and hemorrhage were lessened (Figure 5K). In the high-dose group, some glomeruli in the kidneys were swollen or pyknotic, and the lesions of renal tubular were consistent with the middle-dose group. Interstitial hyperemia and hemorrhage were evident, and there were tubular casts in the remaining tubular (Figure 5L).

## Discussion

Aconite is widely distributed, especially in the Xinjiang Autonomous Region, causing a large amount of economic loss every year. Aconitum alkaloids cause acute poisoning because of its high toxicity (22), however, subacute and chronic poisoning of animals are also common in the grasslands. The animals may display the typical symptoms of aconitine poisoning, such as nausea, vomiting, dizziness, palpitation, hypotension, arrhythmia, and respiratory spasm (3, 23). In our experiments, mice showed varying degrees of poisoning symptoms depending on the dosage. In particular, mice in the high-dose group showed diaphoresis, diarrhea, and obvious neurological symptoms. These results agree with the finding reported by Wada et al. (24), though the symptoms were slightly milder. No mice died during our entire experiment, which could be due to the different dosage of aconitine. The body weight of animals can generally reflect their health status (25). In our study, the treated animals had lower average weights than the control group. This indicates that aconitine can inhibit the growth and development of animals.

As the executor of the animal's physiological function, the organ coefficient can reflect the function and lesion status of the organ (26). A decrease in the organ coefficient indicates that the organ is atrophic and degenerative. An increase in the organ coefficient could reflect the organ's changes of congestion, edema, and hyperplasia (27). The organ coefficient of the liver, spleen, and brain all significantly increased, and the lungs and cerebellar organ coefficient were decreased, suggesting that aconitine can inhibit the growth and development of mice while causing damage to organs.

Physiological and biochemical indices of blood are sensitive indicators of the health of animals, reflecting the metabolism, organ function and nutritional level (28, 29). In this study, both sexes of rats were used in this study, and there was no apparent difference between males and females in the measured parameters. The results of the Hematological indexes showed that the RBC, HGB and HCT in the experimental group were significantly decreased with a time and dose-effect relationship. WBC and Gran in the low-dose group increased, but the middle-dose group and the high-dose group increased first and then decreased. The results indicated that low doses of aconitine improve the body's immune function while high doses are immunosuppressive.

The liver is one of the major target organs of exogenous poisoning. Exogenous toxicants can damage liver cells, and the degree of cellular response can range from mild changes to the cytoplasm to cell death. Measuring the activity of ALT and AST is helpful for determining the extent of liver cell damage (30, 31). Kidneys are the major excretion route of exogenous toxicants; the content of CRE and BUN in serum can be used to evaluate the glomerular filtration, which is of great significance for judging the degree of renal damage (31, 32). We found that with an increase in the time and dose, the activity of ALT and AST increased; the content of CRE and BUN also showed an upward trend with obvious time and dose-effect relationship. This showed that subacute aconitine poisoning can cause various degrees of liver and kidneys functional damage in mice. In addition, the TP and ALB in the mice in each experimental group were significantly decreased. This is commonly seen in malnutrition, protein synthesis and synthesis disorders, functional liver diseases, nephrotic syndrome and other diseases. We demonstrated that aconitine can cause systemic poisoning involving multiple organs and systems in mice.

There are many animal diseases, with complex etiology and pathogenesis. The pathological examination of tissues contributes not only to disease diagnosis but also demonstrating that the poisoning may cause damage (33). Zhu et al. treated Mdr1a^−^/^−^ mice with 0.1 mg/kg aconitine then observed Edema, enlarged tissue space, and karyopyknosis in the hippocampus were observed in the brain, Meanwhile, myocardium fragmentation, deformation, and necrosis of the myocardium were observed in the heart (34). Subsequently, to evaluate the antidotal effect of processed borax against acute and subacute toxicity, cardiac toxicity and neuro-muscular toxicity caused by raw aconite, Sarkar et al. found that the observed changes in aconitine poisoned animals were moderate cell and fluid effusion and severe hemorrhage in lungs (35). Compared with these early studies, similar pathological changes were found in brain, heart and lungs tissues in our experiment. Moreover, there were significantly more megakaryocytes in the spleen of the middle-dose group than the high-dose group. This result was also consistent with the WBCs and Gran alteration in this study. We speculate that middle-dose aconitine may promote immune function in mice, whereas high-doses have significant immunosuppressive effects. These histopathological changes may be the reason for the alteration in the organ coefficient in this study. While combined with the results of hematology, it demonstrates that aconitine could cause chronic hemorrhagic anemia in mice. With the biochemistry detection and previous research results (34–36), we speculate that the target organ of aconitine may be heart, cerebellum, liver, kidney and spleen, which further leads to dysfunction of the systemic organs of mice, causing irreversible toxic damage.

## Conclusion

Our study for the first time explored the effects of subacute aconitine poisoning on hematological and histopathological influences in mice. The results demonstrated that aconitine has marked effects on growth and development in mice, causing systemic poisoning involving multiple organs and systems in a significant time and dose-effect relationship. These results can provide an important theoretical basis for further investigating the mechanism of aconitine toxicity.

## Data Availability Statement

The original contributions presented in the study are included in the article/supplementary material, further inquiries can be directed to the corresponding author.

## Ethics Statement

The animal study was reviewed and approved by the Ethical Principles [Animal (Scientific Procedures) Act 2012] in Animal Research adopted by the China College of Animal Experimentation and were approved by the College of Veterinary Medicine-Northwest A&F University.

## Author Contributions

HL: funding acquisition and writing-original draft. LM, ZG, KW, YZha, ST, YZhu, and BZ: visualization. LM, ZG, KW, YZha, ST, YZhu, HL, and BZ: methodology and writing-review and editing. All authors contributed to the article and approved the submitted version.

## Funding

This work was supported by the grants from the National Natural Science Foundation of China (No. 32072929) and the Science and Technology Special Fund Aid to Qinghai Province (No. 2020-QY-210).

## Conflict of Interest

The authors declare that the research was conducted in the absence of any commercial or financial relationships that could be construed as a potential conflict of interest.

## Publisher's Note

All claims expressed in this article are solely those of the authors and do not necessarily represent those of their affiliated organizations, or those of the publisher, the editors and the reviewers. Any product that may be evaluated in this article, or claim that may be made by its manufacturer, is not guaranteed or endorsed by the publisher.
